# Guava Byproducts (*Psidium guajava* L.) as a Source of Phenolic Compounds with In Vitro Antihyperlipidemic Potential

**DOI:** 10.3390/molecules31101647

**Published:** 2026-05-13

**Authors:** Ramiro Baeza-Jiménez, Juan Antonio Noriega-Rodríguez, Mónica A. Villegas-Ochoa, Gustavo A. Gonzalez-Aguilar, Leticia X. López-Martínez

**Affiliations:** 1 Laboratorio de Biotecnología y Bioingeniería, Centro de Investigación en Alimentación y Desarrollo, A.C. Av. Cuarta Sur 3820, Fracc. Vencedores del Desierto, Delicias 33089, Chihuahua, Mexico; ramiro.baeza@ciad.mx; 2 Departamento de Ingeniería Química y Metalúrgica, Universidad de Sonora, Blvd. Luis Encinas y Rosales s/n, Col. Centro, Hermosillo 83000, Sonora, Mexico; juan.noriega@unison.mx; 3 Coordinación de Tecnología de Alimentos de Origen Vegetal, Centro de Investigación en Alimentación y Desarrollo (CIAD), A. C. Carretera Gustavo Enrique Astiazarán Rosas No. 46, La Victoria, Hermosillo 83304, Sonora, Mexico; mvillegas@ciad.mx; 4 Laboratorio de Antioxidantes y Alimentos Funcionales, SECIHTI-Centro de Investigación en Alimentación y Desarrollo, A.C. Carr. Gustavo Enrique Astiazarán Rosas 46, Col. La Victoria, Hermosillo 83304, Sonora, Mexico

**Keywords:** *Psidium guava*, antioxidant activity, cholesterol, phenolic compounds

## Abstract

Guava (*Psidium guajava* L.) processing produces peels, seeds, and residual pulp. Previous studies have shown that this fraction contains a significant amount of bioactive molecules (more than in the pulp), such as phenolic compounds, which have demonstrated health-beneficial bioactivities, including antioxidant, anti-obesogenic, and antihyperlipidemic activities, among others. In this study, we aimed to characterize total phenolic compounds, total flavonoids, the phenolic profile, and the in vitro antioxidant and antihyperlipidemic properties of guava byproduct extracts, which were evaluated for their potential use as functional ingredients. Phenolic analysis indicated the total phenolic content (46.45 mg GAE/g), the total flavonoid content (3.86 mg CE/g), and contents of individual phenolic compounds, namely ellagic, syringic, rutin, luteolin, and kaempferol. The extracts showed good antioxidant activity (236.22, 344.89, and 387.1 µmol TE/100 g for TEAC, DPPH, and FRAP, respectively). Moreover, the extracts could bind bile acids (18.50–40.34%) and reduce cholesterol solubility in artificial micelles (53.0–67.6%). Modest pancreatic lipase inhibition (33.33%), stronger cholesterol esterase inhibitory activities (60.22%), and mild HMG-CoA inhibitory potential (16.90%) were recorded. Guava processing byproducts demonstrate the potential to be considered as value-added ingredients in functional foods.

## 1. Introduction

Hyperlipidemia is a set of metabolic disorders characterized by high levels of triglycerides and cholesterol in plasma, and these disorders significantly increase the risk of heart disease and stroke [1]. The risk of experiencing these pathological alterations can be reduced by maintaining blood cholesterol within the normal range. Approaches to reduce blood cholesterol have focused on inhibiting endogenous cholesterol synthesis or its absorption [2]. Generally, cholesterol absorption inhibitors can function as bile acid sequestrants and as disruptors of cholesterol micellization, which can be one of the a useful strategy to reduce cholesterol absorption [3,4]. Another important strategy may be inhibiting the action of enzymes such as cholesterol esterase (which hydrolyzes cholesterol esters into cholesterol and free fatty acids before absorption) and pancreatic lipase (which hydrolyzes triglycerides into free fatty acids ready for absorption) [5], as well as 3-hydroxy-3-methylglutaryl coenzyme A (HMG-CoA) reductase (which reduces cholesterol biosynthesis in the liver) [6]. Since intestinal epithelial cells are not capable of directly absorbing triglycerides and cholesterol esters, the role that these enzymes play in absorption is crucial, so their inhibition would enable a reduction in circulating lipid levels and the risk of cardiovascular disease. Several studies have shown that legumes and medicinal plants exert antihyperlipidemic effects due to their bioactive compounds, such as bioactive peptides, saponins, triterpenoids, carotenoids, and phenolic compounds [7,8]. In fruits, various studies have shown that phenolic compound concentrations are higher in peels [9,10]. Previous studies have documented that ethanolic extracts of guava and its byproducts can inhibit pancreatic lipase, potentially providing an alternative to synthetic medications and reducing side effects [11]. Guava (*Psidium guajava* L.) is a tropical fruit that is regularly consumed fresh, but it is also used for producing juice, frozen pulp, marmalades, and beverages. It is estimated that 30% of the total volume of guava fruit, consisting of seeds, pulp remnants, and peels, is discarded after processing [12]. Various studies have shown that these byproducts have the potential for reutilization due to their high phenolic content, which is usually greater than that present in their pulp [13,14]. Phenolic compounds such as ellagic acid, rutin, and kaempferol identified in guava have shown in vitro antioxidant activity, as well as the ability to ameliorate biological markers associated with hyperlipidemia, such as cholesterol esterase and pancreatic lipase inhibition, as well as HMG-CoA [12,15]. Therefore, this study aimed to characterize the in vitro antihyperlipidemic properties of guava byproducts. This description will permit proposing possible uses for the byproducts rather than discarding them, thus generating additional value for this often-ignored part of the fruit.

## 2. Results and Discussion

### 2.1. Phenolic Content

The TPCs obtained in this study (46.45 mg GAE/g) were higher than those reported by de Oliveira et al. [11] in methanolic extracts of guava byproducts (2.34 mg GAE/g) and in ethanolic extracts of guava waste (8.52 mg GAE/g) [15], but close to that reported by Herrera-González et al. [16] in aqueous acetone extracts of Costa Rican guava press cake (51.7 mg GAE/g). The TFC reported in this study (3.86 mg CE/g) is higher than that reported in [17] for guava press cake (1.5 mg CE/g). In contrast, it is lower than the values reported for guava waste by Danielski and Shahidi (4.92 mg CE/g) [15]. Values for TPC and TFC reported across studies can be related to the guava varieties investigated, guava ripening state, drying technique, extraction methods, and specific extraction conditions, including the solvent used. Previous studies have shown that the effectiveness of phenolic compound extraction depends on the type of solvent used and the solubility of the phenolic compounds in the solvent; furthermore, solvent polarity plays an important role in increasing phenolic solubility [18,19]. Then, the use of solvents with different polarities will result in variations in the phenolic compound content of the extracts.

Therefore, it is hard to develop a standard extraction procedure suitable for the extraction of all plant phenols.

**Figure 1:**
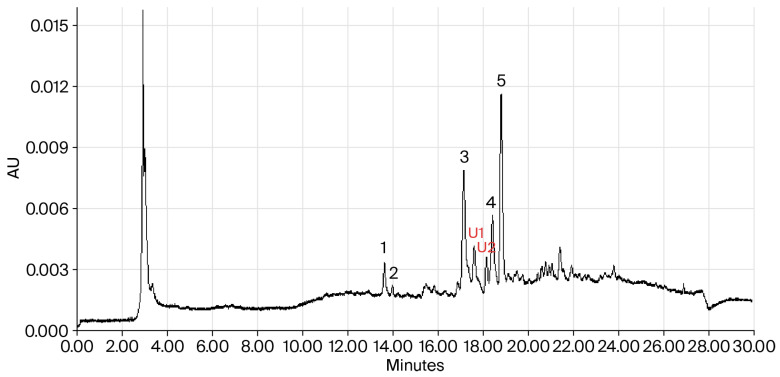
The representative UPLC-DAD chromatogram used to identify and quantify individual phenolic compounds present in guava byproduct methanolic extract: (1) ellagic acid, (2) syringic acid, (3) rutin, U1 (Unidentified 1), U2 (Unidentified 2), (4) luteolin, and (5) kaempferol.

The individual phenolics identified in BPGE (byproduct powders of guava extraction) (Figure 1) are ellagic acid (0.295 µg/g), syringic acid (0.130 µg/g), rutin (40 µg/g), luteolin (22.2 µg/g), and kaempferol (61.6 µg/g). The phenolic profile reported in this study is similar to that reported by Irondi et al. [12] for the methanolic extracts of guava bark steam, but the concentrations differ. The authors identified ellagic acid (3.96 µg/g), rutin (1.37 µg/g), and luteolin (2.83 µg/g). In ripe red guava waste, Danielski and Shahidi [15] identified syringic acid and ellagic acid, findings similar to those of this study. Da Silva Lima et al. [14] determined 47 phenolics in ethanolic extracts of red guava variety “Pedro Sato” waste, including ellagic acid (16,000 µg/g), syringic acid (32 µg/g), and kaempferol (1.66 µg/g), which were also reported in this study. The studies evaluating the phenolic compound profile of guava waste extracts mostly report ellagic acid, luteolin, and kaempferol as the major compounds [14,15].

Despite the substantial levels of these phenolic compounds recorded in this study, the primary phenolic compound detected was kaempferol. This behavior could mainly be due to the extraction method used, the use of different solvents and concentrations, and the methodology used for the identification and quantification of phenolic compounds.

### 2.2. In Vitro Antioxidant Activity

Due to the presence of phenolic compounds in BPGE, they are expected to possess antioxidant capacity. In this study, they were determined in µmol TE/100 g as follows: DPPH (236.22 ± 9.54), TEAC (344.89 ± 11.76), and FRAP (387.71 ± 10.11). Other authors have measured the antioxidant capacity of guava waste, such as De Oliveira et al. [11], who reported 460 and 1096.67 µmol TE/100 g for the TEAC and FRAP methods, respectively, using freeze-dried waste. In another study, Herrera-Gonzalez [16] analyzed ethanolic extracts of fresh guava press cake; they reported values of 271.5, 209.3, and 284.7 µmol TE/g for the DPPH, TEAC, and FRAP assays, respectively, values higher than those found in our study. These results may be due to the use of different waste treatment methods (in our study, the byproducts were dried in an oven), which can degrade sensitive phenolic compounds and affect extraction.

The antioxidant activity of phenolic compounds is related to their chemical structure and capacity to act as radical scavenging agents [20]. The phenolic profile found in BPGE may partially explain the capacity of these byproducts to show radical scavenging activity in the antioxidant assays. It has previously been reported that the free hydroxyl group in phenolic compounds is the main source of antioxidant activity; the greater the number of free hydroxyl groups, the stronger the antioxidant activity, which may be the cause of the strong correlation between phenolic compounds and antioxidant activity [21].

BPGE showed the presence of luteolin and kaempferol flavonoids, relying on the process of donating hydrogen atoms to free radicals to terminate chain reactions [21]. The presence of *ortho*-dihydroxy substitution in the B-ring stabilizes the radical form and participates in electron delocalization; there is also a 2,3-unsaturation and a 4-carbonyl group in the C-ring. These structural characteristics are required for maximum radical scavenging activity in flavonoids [22]. However, kaempferol (3,5,7,4′-OH), in spite of bearing *ortho*-dihydroxy substitution in the B-ring, has high antioxidant activity against some oxidants. This fact can be attributed to the presence of both the 2,3-double bond and the 3-hydroxyl group, meaning that the basic structure of flavonoids becomes important when the antioxidant activity of the B-ring is small [23]. However, BGPE may contain other compounds that can influence antioxidant activity. More research should be conducted to determine which compounds are responsible for the antioxidant activity in the assays evaluated in this study.

### 2.3. Solubility of Cholesterol in Micelles

Cholesterol penetrating the human intestine must be solubilized in micelles to be absorbed. Decreasing cholesterol solubility in micelles can successfully inhibit its absorption and digestion, thereby achieving lipid-lowering effects [24,25].

In Table 1, it is pointed out that the inhibition of cholesterol micellization is induced by BPGE at different concentrations. Such inhibition is concentration-dependent, with mild-to-moderate inhibitory activity ranging from 3.65 to 33.98%. In this context, the presence of phenolic compounds in fruit pulp and peel extracts has demonstrated their ability to inhibit the formation of the micellar structure, essential for cholesterol absorption in the intestine. Ngamukote et al. [26] demonstrated that phenolics in fruit byproducts (grape seeds) can inhibit cholesterol micellization by 50% at a concentration of 0.1 mg/mL. The authors suggest that this inhibition could be linked to the fact that phenolic compounds can bind to bile acids, decreasing the formation of micelles generating structural modifications in micellar cholesterol, which is necessary to dissolve and absorb cholesterol. Results similar to those of this study were reported in [5], whose authors found that grape seed extracts rich in flavonoids inhibited cholesterol micelle formation. According to [3], the interaction between phenolic compounds that occurs in aqueous extracts of bitter melon increases micelle size, leading to the coprecipitation of the aqueous extract with cholesterol, altering the structure of the micelle, as the phenolic compounds decrease the micelle’s ability to incorporate cholesterol.

Although extracts rich in phenolic compounds inhibit cholesterol formation, this inhibition is mild to moderate, suggesting that other compounds, such as saponins, triterpenoids, and phytosterols, present in plant extracts can inhibit micellization [17,27].

### 2.4. Bile Acid Binding Capacity

To evaluate the effect of BPGE on the entrapment capacity of bile acids, its affinity for primary bile acids (taurocholic) and secondary bile acids (taurodeoxycholic and glycodeoxycholic) was determined. As shown in Table 1, the extracts exhibited the concentration-independent binding capacity, with the highest affinity recorded for taurodeoxycholic acid, followed by taurocholic acid and finally glycodeoxycholic acid, at the minimum and maximum tested concentrations (0.25 and 3.0 mg/mL, respectively). At the highest tested dose (3 mg/mL), cholestyramine (a bile acid sequestrant) bound 52.34% to glycocholic acid, 42.70% to taurodeoxycholic acid, and 27.7% to taurocholic acid. It has been proposed that bile acid binding to bioactive compounds, such as phenolic compounds, generates insoluble complexes in the intestine, and these complexes cannot be absorbed, forcing the liver to use endogenous cholesterol to synthesize new bile acids, thereby reducing blood cholesterol levels [28]. BPGE demonstrated the ability to bind both primary and secondary bile acids, regardless of concentration. A behavior similar to that observed in our results was previously reported by [29]; the authors hypothesized that the absence of a concentration-dependent effect could be due to the saturation of bile acid binding sites by phenolic compounds in the aqueous extracts of basil flowers (*Ocimum sanctum* L.). Previous studies have shown the effects of plant extracts and fruit byproducts on the ability to capture bile acids via in vitro models, suggesting that the binding is due to their phenolic compound content [30,31,32].

### 2.5. Enzymatic Inhibition

The inhibition of enzymes responsible for the synthesis of metabolites that contribute to metabolic diseases has emerged as a significant criterion for evaluating the potential health benefits of phenolic compounds derived from plant-based foods [33,34]. Medical drugs play an important role in the management and control of symptoms, but they are also associated with adverse effects. Thus, finding natural-origin inhibitors that also have antioxidant properties is a viable alternative to synthetic drugs.

**Figure 2:**
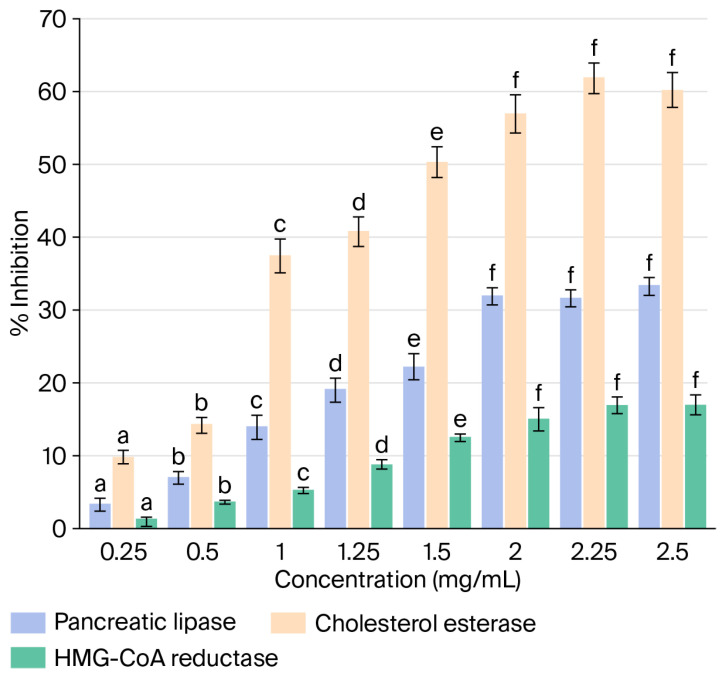
The inhibitory activity of pancreatic lipase, cholesterol esterase, and HGM-CoA reductase from methanolic extracts of guava byproducts. Different letters above the bars indicate significant differences (*p* < 0.05).

In this study, we displayed that guava byproduct extract inhibited pancreatic lipase, cholesterol esterase, and HMG-CoA reductase in a dose-dependent manner (Figure 2). Studies on the inhibition of these enzymes have been extensively conducted on medicinal plant extracts [35,36]. However, research on fruit byproducts is scarce.

#### 2.5.1. Pancreatic Lipase

Pancreatic lipase is the enzyme responsible for the hydrolysis of lipids into monoacylglycerols and free fatty acids in the intestine [37]; its inhibition may have beneficial effects on hyperlipidemia. The extracts evaluated in this study presented inhibitory activity of 33.3% at the highest concentration examined (2.5 mg/mL) and IC_50_ = 2.41 mg/mL. Kaempferol, luteolin, and ellagic acid, as identified in this study, have demonstrated lipase inhibitory activity [38,39] and may be responsible for the observed behavior. However, BGPE may contain other compounds capable of influencing inhibitory activity or synergistic action between flavonoids—flavonoids, flavonoids-phenolic acids that could generate effect on pancreatic lipase inhibition activity [40].

The values of pancreatic lipase inhibition obtained in this study are lower than those described in ethanolic extracts of guava byproducts (IC_50_ = 0.02 mg/mL) [11] and methanolic extracts of steam guava bark, recording values of IC_50_ = 0.022 mg/mL [12], but they are higher than the values reported for ethanolic extracts of guava peel (IC_50_ = 3.50 mg/mL) [9]. Orlistat achieved an inhibition of 87.4% (IC_50_ = 0.30 mg/mL).

#### 2.5.2. Cholesterol Esterase

Under conditions of hyperlipidemia, it is vital to inhibit the generation of cholesterol; one way of doing so is to inhibit cholesterol esterase, which hydrolyzes cholesterol esters into free cholesterol and fatty acids [38,39]. The methanolic extracts of guava byproducts contain varying amounts of phenolic compounds, such as ellagic acid, rutin, and luteolin (Figure 1), which could play a significant role in inhibiting cholesterol esterase activity [41]. The extracts evaluated in this study showed 60.22% inhibitory activity at the maximum concentration tested (2.5 mg/mL) and an IC_50_ of 1.21 mg/mL, close to the level of inhibition identified for simvastatin (74.21%, Ic_50_ = 1.72 mg/mL).

Similarly, ethanolic extracts of passion fruit peel showed 70% inhibition of cholesterol esterase and an IC_50_ value of 0.34 mg/mL [42]. On the other hand, methanolic extracts of Kardan (*C. laurifolius*), a medicinal plant rich in ellagic acid and kaempferol, demonstrated 63.70% inhibition and an IC_50_ of 0.089 mg/mL. The authors assumed that the phenolic compounds identified in the extracts, such as ellagic acid and kaempferol, were responsible for the inhibitory activity [43]. This result represents a greater number of active extracts than reported in our study. Although the reported phenolic compounds identified in BGPE slightly inhibit the cholesterol esterase activity, other compounds such as unidentified compounds (Figure 1) detected in the extract used in this study could influence this activity [30,31].

#### 2.5.3. HMG-CoA Reductase

Under conditions of high blood cholesterol, the inhibition of the HMG-CoA reductase enzyme is a strategic objective for lowering cholesterol levels. In this study, BPGE exhibited a slight, concentration-dependent inhibitory effect (16.9%, IC_50_ > 2.5 mg/mL) on HMG-CoA reductase, while the standard drug pravastatin exhibited an IC_50_ value of 0.053 mg/mL and showed 75.1% enzyme inhibition. In another study, Irondi et al. [12] evaluated methanolic extracts of guava stem bark, reporting an inhibitory activity of 52.3% at the highest concentration tested (0.04 mg/mL), with IC_50_ = 0.032 mg/mL. Ademosun et al. [41] reported that 80% acetone extracts of grapefruit peels showed the capacity to inhibit HMG-CoA reductase (80% at 0.125 mg/mL) with IC _50_ = 0.115 mg/mL. Inhibition studies on fruit byproducts have demonstrated greater activity than those reported in our study.

The proposed mechanism is that phenolic compounds bind to the enzyme in a non-competitive manner or sterically disrupts HMG-CoA binding, blocking the production of mevalonate and thus reducing cholesterol production [44].

The results described in this study indicate a potential use of BPGE for enzyme inhibition. However, further studies must be conducted to validate this observation.

#### 2.5.4. Limitations of the Study

The study focused on only one extraction approach (methanol:water), which limited the exploration of alternative solvents or methods that might recover a broader range of phenolic compounds. Not all the peaks detected by BPGE in the UPLC determination were identified; other detection systems like HPLC-MS allow a wider identification range of phenolic compounds. Regarding the antihyperlipidemic potential of BPGE, transmission electron microscopy could be used to visualize the micellar structures formed during the interaction between BPGE and cholesterol micelles; however, the mechanisms underlying the interaction between phenolic compounds and cholesterol micelles still need to be established.

Finally, in vitro assays employing isolated enzymes may not precisely reflect human physiological conditions, so validation through in vivo models and clinical trials are necessary to confirm the therapeutic potential.

## 3. Materials and Methods

### 3.1. Vegetable Materials

Ripe guavas (*Psidium guajava* L.) were obtained from a local market in Hermosillo, Mexico. The fruits were processed using an electric juice extractor (model 753, Moulinex, Barcelona, Spain) to separate the pulp from the processing byproduct (peels, residual pulp, and crushed seeds). The byproduct fractions were dried at 45 °C for 12 h in an air-circulation oven (FD-23, Binder GmbH, Tuttlingen, Germany). Dry byproducts were ground to obtain a fine powder. The dry byproduct powder of guava (BGP) was stored in amber bags until analysis.

### 3.2. Dry Byproduct Powders of Guava Extraction (BGPE)

#### 3.2.1. Extract Preparation

The extracts were obtained according to the method established in [45]. In brief, 1 g of BPG was homogenized in 20 mL of a methanol/water (80:20, *v*/*v*) solution and sonicated (Branson Ultrasonic Co., Danbury, CT, USA) for 30 min. After this, the samples were centrifuged (Allegra 64R Centrifuge, Beckman Colter, Indianapolis, IN, USA) at 9000 rpm at 4 °C for 15 min. Supernatants were collected, and the residues were re-extracted under similar conditions. Supernatants were combined and filtered through Whatman^®^ no. 1 filter paper, (Cytiva, Maidstone, UK) and the filtrate was utilized to determine the total phenolic content, total flavonoid content, and phenolic composition. To determine enzyme inhibition caused by the extracts, the filtrate was rotary evaporated at 40 °C to remove methanol, and the water was freeze-dried. Dried BPGE was redissolved in sodium phosphate buffer (0.1 M, pH 6.9) at the time of analysis.

#### 3.2.2. Determination of the Total Phenolic Content (TPC)

TPC was determined using the method of Singleton et al. [46], with some modifications. The absorbance was measured at 765 nm using a microplate reader (FLUOstar Omega, B.M.G. Labtech, Durham, NC, USA). TPC was expressed as mg of gallic acid equivalent per 100 g (mg GAE/ g).

#### 3.2.3. Determination of the Total Flavonoid Content (TFC)

TFC was determined using the spectrophotometric assay established in [47]. Absorbance was measured at 510 nm. Flavonoid content was expressed as mg catechin equivalents (CE)/g.

#### 3.2.4. Determination of Individual Phenolic Compounds

The individual phenolic compounds of BPGE were estimated via the method of Velderrain-Rodríguez et al. [48] using a UPLC system (Acquity, Waters Co., Milford, MA, USA) with a photodiode array detector. The individual phenolic compounds were identified by matching their retention times and absorption spectra with their respective standards, and their contents were estimated based on calibration curves. The results were expressed as μg/g of BPGP.

### 3.3. Determination of Antioxidant Capacity

Antioxidant capacity was determined using three different assays: Trolox equivalent antioxidant capacity (TEAC), as described by Re et al. [49]; 2,2-diphenyl-1-picrylhydrazyl (DPPH), as established by Brand-Williams et al. [50]; and ferric reducing antioxidant power (FRAP), as described by Benzie and Strain [51]. Assays were performed in microplate wells, and absorbance was measured using a microplate reader. The results for antioxidant capacity were expressed as µmol TE/100 g.

### 3.4. Determination of Bile Acid Binding Capacity

The entrapment capacity of BPGE with primary bile acids (taurocholic) and secondary bile acids (taurodeoxycholic and glycodeoxycholic) was evaluated using the method proposed by Lin et al. [52]. The free bile acids in the supernatant were determined using a total bile acids kit (Cosmo Bio Co., Ltd., Tokyo, Japan) according to the manufacturer’s instructions. Cholestyramine (3 mg/mL) was used as a control. The ability to trap bile acids was reported as the bile acid binding percentage.

### 3.5. Inhibition of Cholesterol Micellization

The artificial micelles were prepared based on the description in [52]. The amount of cholesterol in the supernatant (representing micellar cholesterol) was determined using a total cholesterol assay kit (Lab Assay™ Cholesterol Kit, Wako, Osaka, Japan), according to the manufacturer’s instructions. The results were expressed as the percentage of inhibition of cholesterol micellization.

### 3.6. Determination of Enzymatic Inhibition

#### 3.6.1. Pancreatic Lipase Inhibition Assay

The pancreatic lipase inhibitory activity of BPGE was evaluated using the method of Worsztynowicz et al. [53]. The results were expressed as a percentage of lipase inhibition. Orlistat was used as a positive control (0.25–2.5 mg/mL).

#### 3.6.2. Inhibition of Cholesterol Esterase

The inhibition of cholesterol esterase activity was determined according to the method established by Pietsch and Gutschow [54]. The absorbance at 405 nm was determined using a microplate reader. Simvastatin (0.25–2.5 mg/mL) was used as a positive control. The results were expressed as a percentage of cholesterol esterase inhibition.

#### 3.6.3. HMG-CoA Reductase Inhibition Activity

The inhibitory activity of BPGE on HMG-CoA reductase was determined using an HMG-CoA reductase assay kit (Sigma-Aldrich Co., St Louis, MO, USA) according to the manufacturer’s recommendations. Simvastatin was used as a positive control. HMG-CoA reductase inhibition was reported as a percentage of HMG-CoA inhibition.

The enzymatic inhibition percentages were calculated according to the following formula:

%Inhibition = [(A_blank_ − A_extract_)/A_blank_] × 100

where A_blank_ is the absorbance of the blank (mixture without extract) and A_extract_ is the absorbance of the mixture with extract.

In addition, IC_50_ values were obtained from the regression of the logarithm of the extract concentrations versus inhibitory activity (%).

## 4. Statistical Analysis

Experiments were conducted in triplicate, and the results are presented as mean ± standard deviation (SD). Analysis of variance (ANOVA) and Tukey’s test were performed (*p* < 0.05) to determine statistical differences. Data were analyzed using NCSS 2012 statistical analysis software version: 12.0.17 (NCSS LLC, Kaysville, UT, USA).

## 5. Conclusions

In this study, we show the potential application of a byproduct usually discarded as agro-industrial waste. For the methanolic byproduct of guava extract, kaempferol and ellagic acid were the most abundant phenolic compounds, and they are capable of delaying the digestion and absorption of fats by inhibiting the activity of pancreatic lipase, cholesterol esterase, and HMG-CoA enzymes in vitro in a dose-dependent manner, as well as moderately inhibiting cholesterol micellization and bile acid binding. The extract also exhibited antioxidant activity, as measured via the DPPH, TEAC, and FRAP scavenging assays. These bioactivities are due to the presence of bioactive compounds in the extracts, such as phenolic compounds, which demonstrate the significant health benefits of revaluing these byproducts as functional ingredients in the treatment of hyperlipidemia. Additional research is needed on the contents of other bioactive molecules in fruit byproducts, such as guava, as well as on their potential mechanisms of action.

## Figures and Tables

**Table 1:** Cholesterol micellization and the bile acid binding capacity of methanolic extracts of guava byproducts.

		Bile Acid Binding (%)		
Concentration (mg/mL)	Cholesterol Micellization Inhibition (%)	Taurocholic Acid	Taurodeoxycholic Acid	Glycodeoxycholic Acid
0.5	15.63 ± 1.21 ^e^	22.43 ± 0.93 ^e^	25.11 ± 1.11 ^e^	18.59 ± 1.16 ^e^
1.0	20.22 ± 1.33 ^d^	24.78 ± 1.66 ^d^	33.19 ± 2.05 ^d^	20.01 ± 1.63 ^d^
1.5	25.53 ± 1.34 ^c^	27.11 ± 2.03 ^c^	35.30 ± 1.17 ^c^	24.61 ± 0.88 ^c^
2.0	33.44 ± 2.11 ^ba^	34.11 ± 1.11 ^b^	38.37 ± 1.22 ^b^	27.43 ± 1.66 ^b^
2.5	35.89 ± 1.78 ^a^	37.32 ± 1.43 ^a^	40.03 ± 3.44 ^a^	31.12 ± 1.71 ^a^
3.0	35.26 ± 2.10 ^a^	37.69 ± 1.70 ^a^	40.34 ± 2.17 ^a^	31.67 ± 2.02 ^a^

The data are expressed as mean and standard deviation (*n* = 3). The values in the same row with different superscript letters differ significantly (*p* < 0.05).

## Data Availability

Data are contained within the article/Supplementary Material.

## Supplementary Materials

The following supporting information can be downloaded at: https://www.mdpi.com/article/10.3390/molecules31101647/s1, File S1: Methodology.

## Abbreviations

The following abbreviations are used in this manuscript:

TPC	Total phenolic content
TFC	Total flavonoid content
BPG	Byproduct powders of guava
BPGE	Byproduct powders of guava extraction
UPLC	Ultra-high-performance liquid chromatography
DPPH	1,1-diphenyl-2-picrylhydrazyl
TEAC	Trolox equivalent antioxidant capacity
FRAP	Ferric reducing antioxidant power
HMG-CoA	Enzyme 3-hydroxy-3-methylglutaryl coenzyme
Ic_50_	Half maximal inhibitory concentration
