# Supplementary material for: Guava Byproducts (Psidium guajava L.) as a Source of Phenolic Compounds with In Vitro Antihyperlipidemic Potential
Source: Molecules. 2026 May 13;31(10):1647. doi: 10.3390/molecules31101647 (PMC13209802; doi:10.3390/molecules31101647)
Supplement: Supplementary file 1 [file molecules-31-01647-s001.zip › molecules-4275808-File S1.pdf]

Article

Guava Byproducts (*Psidium guajava* L.) as a Source of Phenolic Compounds with In Vitro Antihyperlipidemic Potential

Ramiro Baeza-Jiménez [1], Juan Antonio Noriega-Rodríguez [2], Mónica A. Villegas-Ochoa [3], Gustavo A. Gonzalez-Aguilar [3,*] and Leticia X. López-Martínez [3,4,*]

[1] Laboratorio de Biotecnología y Bioingeniería, Centro de Investigación en Alimentación y Desarrollo, A.C. Av. Cuarta Sur 3820, Fracc. Vencedores del Desierto, Delicias 33089, Chihuahua, Mexico; ramiro.baeza@ciad.mx
[2] Departamento de Ingeniería Química y Metalúrgica, Universidad de Sonora, Blvd. Luis Encinas y Rosales s/n, Col. Centro, Hermosillo 83000, Sonora, Mexico; juan.noriega@unison.mx
[3] Coordinación de Tecnología de Alimentos de Origen Vegetal, Centro de Investigación en Alimentación y Desarrollo (CIAD), A. C. Carretera Gustavo Enrique Astiazarán Rosas No. 46, La Victoria, Hermosillo 83304, Sonora, Mexico; mvillegas@ciad.mx
[4] Laboratorio de Antioxidantes y Alimentos Funcionales, SECIHTI-Centro de Investigación en Alimentación y Desarrollo, A.C. Carr. Gustavo Enrique Astiazarán Rosas 46, Col. La Victoria, Hermosillo 83304, Sonora, Mexico
* Correspondence: gustavo@ciad.mx (G.A.G.-A.); leticia.lopez@ciad.mx (L.X.L.-M.)

3. Materials and Methods

3.1. Vegetable materials

Ripe guavas (*Psidium guajava* L.) were obtained from a local market in Hermosillo, Mexico. The fruits were processed using an electric juice extractor (model 753, Moulinex, Spain) to separate the pulp from the processing byproduct (peels, residual pulp, and crushed seeds). The byproduct fractions were dried at 45 °C for 12 h in an air-circulation oven (FD-23, Binder GmbH, Tuttlingen, Germany). Dry byproducts were ground to obtain a fine powder. The dry byproduct powder of guava (BGP) was stored in amber bags until analysis.

3.2. Dry byproduct powders of guava extraction (BGPE)

3.2.1. Extract preparation

The extracts were obtained according to the method established in [45]. In brief, 1 g of BPG was homogenized in 20 mL of a methanol/water (80:20, v/v) solution and sonicated (Branson Ultrasonic Co., Danbury, CT, USA) for 30 min. After this, the samples were centrifuged (Allegra 64R Centrifuge, Beckman Colter, Indianapolis, IN, USA) at 9000 rpm at 4°C for 15 min. Supernatants were collected, and the residues were re-extracted under similar conditions. Supernatants were combined and filtered through Whatman® no. 1 filter paper, and the filtrate was utilized to determine the total phenolic content, total flavonoid content, and phenolic composition. To determine enzyme inhibition caused by the extracts, the filtrate was rotary evaporated at 40°C to remove methanol, and the water was freeze-dried. Dried BPGE was redissolved in sodium phosphate buffer (0.1 M, pH 6.9) at the time of analysis.

3.2.2. Determination of the total phenolic content (TPC)

TPC was determined using the method of Singleton et al. [46], with some modifications. The BGEP (15 µL) was mixed with 15 µL of 2 M Folin–Ciocalteu reagent and 240 µL of distilled water in a 96-well microplate and mixed slightly for 3 min, and then 30 µL of 2 M Na_2CO_3 was added; microplates were then allowed to stand without light for 90 min. The absorbance was measured at 765 nm using a microplate reader (FLUOstar Omega,

B.M.G. Labtech, Durham, NC, USA). TPC was expressed as mg of gallic acid equivalent per 100 g (mg GAE/ g).

3.2.3. Determination of the total flavonoid content (TFC)

TFC was determined using the spectrophotometric assay established in [47]. In brief, 50 µL of BGEP was mixed with 320 µL of distilled deionized water. At time zero, 150µL of 5% (w/v) sodium nitrite ($NaNO_2$) solution was added and mixed. After 5 min, 150 µL of 10% (w/v) aluminum chloride ($AlCl_3$) was added and mixed. After 6 min, 1 mL of 1 M sodium hydroxide (NaOH) was added and mixed. Absorbance was measured at 510nm. Flavonoid content was expressed as mg catechin equivalents (CE)/g.

3.2.4. Determination of individual phenolic compounds

The individual phenolic compounds of BPGE were estimated via the method of Velderrain-Rodríguez et al. [48] using an UPLC system (Acquity, Waters Co., Milford, MA, USA) with a photodiode array detector. Separation was performed using an UPLC BEH C18 column (1.7 µm, 3.0×100 mm). The mobile phase was A (0.1%, *v/v*, acetic acid in water) and B (0.1%, *v/v*, acetic acid in methanol) at a flow rate of 0.7 mL/min. The solvent gradient was initially 91% of A and 9% of B (0–11 min), 9% to 14% of B (11–15 min), and 15% of B (10 min). The individual phenolic compounds were identified by matching their retention times and absorption spectra with their respective standards, and their contents were estimated based on calibration curves. The results were expressed as µg/g of BPGP.

3.3. Determination of antioxidant capacity

Antioxidant capacity was determined using three different assays: Trolox equivalent antioxidant capacity (TEAC), as described by Re et al. [49]; 2,2-diphenyl-1-picrylhydrazyl (DPPH), as established Brand-Williams et al. [50]; and ferric reducing antioxidant power (FRAP), as described Benzie and Strain [51]. Assays were performed in microplate wells, and absorbance was measured using a microplate reader. The results for antioxidant capacity were expressed as µmol TE/100 g.

3.4. Determination of Bile Acid Binding Capacity

The entrapment capacity of BPGE with primary bile acids (taurocholic) and secondary bile acids (taurodeoxycholic and glycodeoxycholic) was evaluated using the method proposed by Lin et al. [52]. In brief, 100 µL of BPGE (0.5-3.0 mg/mL) was mixed with 900 µL of bile acids at 2 mM in 0.1 M phosphate-buffered saline, pH 7, and incubated for 90 min at 37 °C. The mixtures were centrifuged at 11,000 rpm for 15 min. The free bile acids in the supernatant were determined using a total bile acids kit (Cosmo Bio Co. Ltd., Tokyo, Japan) according to the manufacturer's instructions. Cholestyramine (3 mg/mL) was used as a control. The ability to trap bile acids was reported as the bile acid binding percentage.

3.5. Inhibition of cholesterol micellization

The artificial micelles were prepared based on the description in [52]. Mixtures of 2 mM cholesterol, 1 mM oleic acid, and 2.4 mM phosphatidylcholine were dissolved in methanol and dried under a nitrogen stream; subsequently, a 15 mM saline phosphate buffer (pH 7.4) containing 6.6 mM taurocholate salt was added. The suspension was sonicated twice at 25 kHz for 30 min to induce micelle formation. The micelle solutions were incubated for 8 hours at 37 °C. A total of 25 µL BPGE (ranging from 0.5 to 3.0 mg/mL) or 25 µL of phosphate-buffered saline (as a control) were added to the micelle solutions and incubated for an additional 2 h at 37 °C. Subsequently, the solutions were centrifuged at 16,000 rpm for 20 minutes at 25 °C. The amount of cholesterol in the supernatant (representing micellar cholesterol) was determined using a total cholesterol assay kit (Lab Assay™ Cholesterol Kit, Wako), according to the manufacturer's instructions. The results were expressed as the percentage of inhibition of cholesterol micellization.

3.6. Determination of Enzymatic Inhibition

3.6.1. Pancreatic lipase inhibition assay

The pancreatic lipase inhibitory activity of BPGE was evaluated using the method of Worsztynowicz et al. [53]. In brief, 20 μL of peel powder extracts (0.25 to 2.5 mg/mL) and 20 μL of a pancreatic lipase solution (1 mg/mL) (100–500 U/mg) were incubated at 37°C for 10 min; then, 1.8 mL of a cholate solution in 0.1 M sodium phosphate buffer (1.15 mg/mL) containing gum Arabic (0.55 mg/mL) and 20 μL of *p*-nitrophenyl palmitate (0.01 M) in isopropanol were added. The mixture was incubated at 37°C for 10 min. The released *p*-nitrophenyl was determined at 410 nm using a microplate reader after 10 min of incubation at 37°C. The results were expressed as a percentage of lipase inhibition. Orlistat was used as a positive control (0.25-2.5 mg/mL).

3.6.2. Inhibition of cholesterol esterase

The inhibition of CEasa activity was determined according to the method established by Pietsch and Gutschow [54]. A volume of 50 μL of BPGE, at concentrations ranging from 0.25 to 2.5 mg/mL, was incubated with a mixture of 100 μL of taurocholic acid in 5.16 mM of 0.1 M sodium phosphate buffer and 90 μL of 0.2 mM p-nitrophenylbutyrate (in sodium phosphate buffer, pH 7.0, diluted with 100 mM NaCl). The reaction was initiated by adding 50 μL of pancreatic cholesterol esterase (1 mg/mL in phosphate buffer, pH 7.0) to the mixture. After incubating for 5 min at 25 °C, the absorbance at 405 nm was determined using a microplate reader. Simvastatin (0.25-2.5 mg/mL) was used as a positive control. The results were expressed as a percentage of CEase inhibition.

3.6.3. HMG-CoA reductase inhibition activity

The inhibitory activity of BPGE on HMG-CoA reductase was determined using an HMG-CoA reductase assay kit (Sigma-Aldrich Co., St Louis, MO, USA) according to the manufacturer's recommendations. The reaction mixture contained nicotinamide adenine dinucleotide phosphate (NADPH; 400 μM) and HMG-CoA (400 μM) at a final volume of 200 μL of potassium phosphate buffer (100 mM at pH 7.4). The reaction was started by adding 2 μL of HMG-CoA reductase and incubated at 37 °C for 10 min with 2 μL of BPGE (0.25–2.5 mg/mL). Pravastatin was used as a standard. The absorbance of the reaction mixture was measured at 340 nm. The reaction was incubated at 37°C for 10 min, and the absorbance was measured at 340 nm. Simvastatin was used as a positive control. HMG-CoA reductase inhibition was reported as a percentage of HMG-CoA inhibition.

The enzymatic inhibition percentages were calculated according to the following formula:

$\%Inhibition = [(A_{blank}–A_{extract})/A_{blank}] \times 100$

where A_{blank} is the absorbance of the blank (mixture without extract) and $A_{extract}$ is the absorbance of the mixture with extract.

In addition, IC_{50} values were obtained from the regression of the logarithm of the extract concentrations versus inhibitory activity (%).

4. Statistical analysis

Experiments were conducted in triplicate, and the results are presented as mean ± standard deviation (SD). Analysis of variance (ANOVA) and Tukey's test were performed ($p < 0.05$) to determine statistical differences. Data were analyzed using NCSS 2012 statistical analysis software (NCSS LLC, Kaysville, UT, USA).
